# Physiological, Diurnal and Stress-Related Variability of Cadmium-Metallothionein Gene Expression in Land Snails

**DOI:** 10.1371/journal.pone.0150442

**Published:** 2016-03-02

**Authors:** Veronika Pedrini-Martha, Michael Niederwanger, Renate Kopp, Raimund Schnegg, Reinhard Dallinger

**Affiliations:** Department of Ecophysiology, Division of Zoology, University of Innsbruck, Innsbruck, Tirol, Austria; CINVESTAV-IPN, MEXICO

## Abstract

The terrestrial Roman snail *Helix pomatia* has successfully adapted to strongly fluctuating conditions in its natural soil habitat. Part of the snail’s stress defense strategy is its ability to express Metallothioneins (MTs). These are multifunctional, cysteine-rich proteins that bind and inactivate transition metal ions (Cd^2+^, Zn^2+^, Cu^+^) with high affinity. In *Helix pomatia* a Cadmium (Cd)-selective, inducible Metallothionein Isoform (CdMT) is mainly involved in detoxification of this harmful metal. In addition, the snail CdMT has been shown to also respond to certain physiological stressors. The aim of the present study was to investigate the physiological and diurnal variability of *CdMT* gene expression in snails exposed to Cd and non-metallic stressors such as desiccation and oxygen depletion. *CdMT* gene expression was upregulated by Cd exposure and desiccation, whereas no significant impact on the expression of *CdMT* was measured due to oxygen depletion. Overall, Cd was clearly more effective as an inducer of the *CdMT* gene expression compared to the applied non-metallic stressors. In unexposed snails, diurnal rhythmicity of *CdMT* gene expression was observed with higher mRNA concentrations at night compared to daytime. This rhythmicity was severely disrupted in Cd-exposed snails which exhibited highest *CdMT* gene transcription rates in the morning. Apart from diurnal rhythmicity, feeding activity also had a strong impact on *CdMT* gene expression. Although underlying mechanisms are not completely understood, it is clear that factors increasing *MT* expression variability have to be considered when using MT mRNA quantification as a biomarker for environmental stressors.

## Introduction

Snails (Gastropoda, Mollusca) have been able to adapt to diverse ecosystems on land and sea, sometimes being exposed to very harsh conditions in their natural habitats. Terrestrial gastropods, in particular, had to evolve survival strategies to cope with fluctuating environmental conditions such as variations of temperature or water supply, and soil mineral availability due to occasionally fast changing microclimatic conditions. One survival strategy of these species to overcome unfavorable weather conditions during summer (e.g. drought), is to change into a state of dormancy, the so-called aestivation. As a consequence, many physiological functions such as the heart rate [1] or the reproductive activity decline [2]. During long-term aestivation, moreover, tolerance to hypoxia and pH disturbances as well as coping with reactive oxygen species to prevent oxidative cell damage, are of vital importance to snails [3,4]. To survive the harsh conditions in winter, when temperature can reach sub-zero levels from September-October until March, snails undergo another kind of dormancy, called hibernation [5,6]. A major difference between these two dormant states is, that hibernation is mainly triggered by the photoperiod [5,7] whereas aestivation is a response to unfavorable environmental conditions in the natural habitat of snails during their active season from March to September-October [6]. During dormancy (aestivation or hibernation), animals enter a hypometabolic state where metabolic rate depression results in silencing or reducing global gene transcription and translation as well as energy consuming biosynthetic processes [8,9]. In addition, the composition of haemolymph is altered [6]. While during hibernation triglyceride and galactose concentrations of the haemolymph increase, aestivating snails accumulate polyols and saccharides in their haemolymph [6].

It is supposed that rhythms of biological activity in snails are mainly influenced by environmental factors, the biological clock and social interactions [5]. The Roman snail *Helix pomatia*, for example, inhabits many terrestrial habitats in central Europe [10], being most active at dawn [11]. Rain, however, can modify its biorhythm towards staying active all day [5,11]. In mammals, a master pacemaker of oscillating nerve cells is present in the suprachiasmatic nucleus to control rhythms during daily activity and rest [12]. Until now, however, no master pacemaker could be identified in helicid snails. Also, there is no or only scarce knowledge about the existence, organisation and function of endogenous oscillators or biological clocks and their interaction with environmental factors in terrestrial gastropods.

A further important task for the viability of soil living snails is their capacity to regulate and excrete metallic trace elements, and to detoxify non-essential harmful metal ions [13]. As a consequence, terrestrial helicid snails have developed the capability to accumulate and tolerate elevated amounts of toxic metals such as Cadmium (Cd) without experiencing, below a certain threshold concentration, major damage to the organism [13–16]. Therefore, *Helix pomatia* serves as a relevant species for monitoring environmental Cd pollution [17–18].

The exceptional tolerance of helicid snails towards Cd accumulation is based on the expression of Metallothioneins (MTs) [13,19]. MTs are multifunctional proteins involved in diverse biological tasks such as metal homeostasis and detoxification [20,21], stress response [22], protection of cells against oxidative stress [23] and gene regulation [24]. Typical features of MT proteins are their low molecular weight, high metal content, low abundance or lack of aromatic amino acids and a high content of cysteines, arranged in characteristic Cys-Xaa-Cys motifs, which are vital to bind mono- and divalent transition metal ions through their sulfhydryl groups within the so-called metal thiolate clusters [25]. Helicid gastropod MTs exhibit a unique feature compared to all other MTs by the evolution of metal-specific MT isoforms, originated from metal-unspecific MT precursors in basal gastropod ancestors [19,26]. The Roman snail *Helix pomatia*, in particular, possesses a copper-specific MT (CuMT), binding Cu^+^ ions with high selectivity [20,27], and a Cd-selective MT (CdMT), predominantly binding Cd^2+^ ions upon metal exposure, as well as Zn^2+^ ions in the absence of Cd stress [13]. These two MT isoforms are differentially expressed in a tissue-specific manner [20,28] and exert differential, metal-specific functions. The CuMT plays an important role in metal homeostasis and hemocyanin synthesis in a cell type called rhogocyte [20,27,29] whereas the CdMT, mainly expressed in hepatopancreatic and digestive tissues, is involved in Cd^2+^ detoxification and stress response [13,15,30]. Due to its specific metal binding and responsiveness towards exposure, the CdMT protein was proposed as a biomarker for environmental metal pollution [17,18]. A third unspecific MT isoform, the so-called Cd/Cu-MT, was detected at very low expression levels. It binds Cd^2+^ and Cu+ ions simultaneously, as previously shown for the near relative *Cantareus aspersus* [31].

There is increasing evidence that the stress defense system of organisms can be influenced by fluctuations of activity or diurnal rhythmicity. For example, protein activity by phosphorylation [32] as well as mRNA expression and protein synthesis can show diurnal expression patterns or circadian rhythmicity [33–36]. This means, that the toxic effect of a stressor to the cell and subsequently the survival of the organism can depend on the day time individuals are exposed to [37]. However, there is scarce data about the influence of diurnal rhythmicity on the response capacity towards stressors in invertebrates and terrestrial snails, in particular. As Cd is concerned, this metal is inactivated in snail tissues by a specific CdMT isoform which selectively binds Cd^2+^ ions with high efficiency [19,20]. On the other hand, the *CdMT* encoding gene seems to respond to environmental stressors less specifically, exhibiting a high degree of plasticity and hence, may allow terrestrial snails to respond to different stressors individually [30].

Against this background, the main purpose of this study was to investigate the transcriptional activity of the *CdMT* gene of *Helix pomatia* in response to natural environmental stressors such as desiccation and oxygen depletion on the one hand, and Cd exposure on the other, by considering factors that may influence individual variability. In addition to this, the potential impact of diurnal rhythmicity on the time course of *CdMT* gene expression in un-stressed and Cd-exposed Roman snails was investigated. This may not only be important for physiological reasons, but may also be relevant in view of using snail MT mRNA quantification in biomarker studies.

## Materials and Methods

### Rearing of snails

Adult Roman snails (*Helix pomatia*) were obtained from a commercial supplier (Thüringer Weinbergschnecke, Germany). Prior to exposure, they were kept for several weeks in groups of up to 50 individuals in large wire mesh-sealed plastic boxes (80 x 30 x 20 cm) on commercial garden soil supplemented with lime powder (CaCO_3_) under constant conditions (18°C, 12 h light/dark cycle– 7 am light on and 7 pm light off). Every two to three days, boxes were cleaned, and animals were fed with salad (*Lactuca sativa*) ad libitum and sprayed with water.

### Exposure experiments

For Cd exposure, snails were transferred to smaller plastic boxes and separated into a control and a metal-treated group with 30 individuals each. Control snails were fed with uncontaminated lettuce (4.41 ± 1.55 μg Cd / g dry wt.; n = 5), whereas animals of the metal-exposed group were supplied with Cd-enriched salad (121.33 ± 50.50 μg Cd / g dry wt.; n = 5) which had previously been incubated with a CdCl_2_ solution (2 mg Cd / L) for one hour [17]. The duration of the experiment was 14 days.

For the diurnal cycle experiment, snails were kept individually in transparent octagonal plastic boxes (diameter: 12 cm; height: 6 cm) on commercial garden soil supplemented with lime powder (CaCO_3_). The light period (day) lasted from 07:00 am to 07:00 pm and the dark period (night) from 07:00 pm to 07:00 am. For acclimatization, animals were fed regularly with salad (*Lactuca sativa*) and sprayed with water for one week. During the experiment, control snails (n = 35) were fed with uncontaminated salad whereas metal-exposed animals (n = 35) were fed with Cd-enriched salad daily as described above for four to five days, depending on the time of sampling (see tissue dissection) until the end of the experiment. Feeding behavior was documented by taking photos of each snail before and after supplying fresh lettuce leaves.

For the oxygen depletion experiment, snails were acclimatized in a sealed transparent acrylic glass chamber at room temperature (RT) for over 12 hours (rel. air humidity approx. 60%). Anoxia was established within 7 to 8 minutes by filling the chamber with nitrogen, to which snails were exposed over a period of 24 hours. Oxygen levels (approx. 0.06%) were measured and regulated by an oxygen probe (PreSense, Regensburg, Germany) throughout the whole experiment. Control snails were held under stable aerated conditions as described above.

For the desiccation experiment, snails were kept in a climate chamber under regular conditions as described above. Controls were fed and sprayed with water every two to three days. Snails of the desiccation group were kept under aestivating conditions with a high aeration rate due to air circulation and without feeding through a period of 21 days.

### Tissue dissection

For the Cd exposure experiment, five individuals of each group were dissected for isolation of midgut gland tissue on days 1, 3, 5, 8 and 14. In addition, five control animals were sampled on day 0. For the circadian experiment, five controls and five Cd-fed individuals were dissected for isolation of midgut gland tissue every four hours starting on day four of the Cd exposure at 04:30 pm until day five at 12:30 pm. Dissection of snails at night (time points 08:30 pm, 12:30 am, 04:30 am) was carried out in the dark under red light. For the oxygen depletion experiment, five controls and five individuals exposed to anoxic conditions were dissected after 12 and 24 hours and midgut gland tissue was isolated. For the desiccation experiment, midgut gland tissue of five controls and five dehydrated snails was isolated after 21 days of exsiccation.

Dissection was carried out on an ice-cooled aluminum plate periodically cleansed with distilled water and RNase AWAY (Sigma-Aldrich). From each individual, small tissue aliquots (1–2 mg fresh wt.) were transferred to RNA Later (ambion®by Life Technologies®) and stored at -80°C until RNA isolation. The remaining midgut gland tissue of each individual was transferred into screw-capped polyethylene tubes (Greiner, Austria) and oven-dried at 60°C.

### RNA isolation, cDNA synthesis and quantitative Real Time PCR

For RNA isolation, tissue aliquots were homogenized in TRIzol reagent (ambion®by Life Technologies®) using the Ultra-Turrax T25 (Janke & Kunkel IKA®Labortechnik). After DNase I digestion (Invitrogen) RNA was cleaned up with the RNeasy MiniElute Kit (Qiagen, Hilden, Germany). Total RNA was quantified using the Quant-iT^TM^ Ribogreen® RNA quantification Kit (Invitrogen). 300 to 450 ng of total RNA were applied to a total volume of 50 μl for cDNA synthesis using the RevertAid^TM^ H Minus M-MLV Reverse Transcriptase (Fermentas).

Quantitative Real Time PCR (qRT-PCR) of the *CdMT* gene of *Helix pomatia* was accomplished using a 7500 Real Time PCR Analyzer with Power SYBR® Green detection (Applied Biosystems). Δct values were assessed by means of calibration curves from amplicon plasmids cloned with the TOPO TA Cloning® Kit for sequencing (Invitrogen). Primers were designed and applied as previously described, using total RNA as a reference for transcriptional quantification [38].

### Sample digestion and metal analysis

Midgut gland tissue samples as well as control and Cd-soaked salad leaves (*Lactuca sativa*) were oven-dried at 60°C. After determination of dry weight (d.w.), samples were heat-digested at 70°C with a mixture (2–3 ml) of nitric acid (suprapur, Merck) and deionized water (1:1) in 12 ml screw-capped polyethylene tubes (Greiner, Austria) for several days until a clear solution was obtained. When necessary, the digestive mixture was renewed from time to time to compensate for evaporation. For complete oxidation, the remaining digestion solution was spiked with a few drops of H_2_O_2_ and diluted with acidified deionized water (5% nitric acid) to a final volume of 11.5 ml.

Cd concentrations were measured by flame (model 2380, Perkin Elmer, Boston, MA) or graphite furnace atomic absorption spectrophotometry with polarized Zeeman background correction (model Z-8200, Hitachi, Japan) and Pd(NO_3_)_2_ as a matrix modifier, depending on concentration levels in the samples. In either case, calibration was performed with diluted titrisol standard solutions (Merck) prepared with de-ionized water and 5% nitric acid (suprapur, Merck). Specimens with lobster hepatopancreas (TORT-2, National Research Council, Ottawa, Canada) were used as standard reference material and processed in the same way as the samples (n = 5). Cd concentrations of the standard reference material were confirmed to be within the accepted deviations (±10%) from certified values.

### Metallothionein quantification

CdMT concentrations in Roman snail midgut gland tissue were not measured directly. Instead, they were estimated based on the known binding stoichiometry between molar ratios of Cd^2+^ and CdMT concentrations in samples of Cd-exposed snail tissues, as already applied in previous studies by means of calibration curves [18]. The estimation works owing to the inducibility of the CdMT by Cd and the established Cd^2+^ binding specificity of the expressed CdMT protein [39].

### Statistics

For statistical analysis of qRT-PCR and Metal measurements, Sigma Plot 12.5 (2011 Systat Software, Inc.) was applied and data were tested for significant outliers (Grubb’s test; P<0.05). Data were proved for homoscedasticity and normal distribution (Shapiro-Wilk). For the oxygen depletion experiment, all data were normally distributed and different groups were compared applying a Two Way ANOVA (P<0.05). Due to very high SDs data derived from the metal measurement of the metal exposure experiment were log transformed. For the metal exposure experiment and the diurnal experiment a Two Way ANOVA of data derived from qRT PCR and metal measurement was performed. Also a multiple comparison procedure comparing the different days / time points (Holm-Sidak method) and differences of controls and Cd exposed individuals of each day / time point (Bonferroni correction) was carried out. For both groups of the circadian experiment, controls and Cd-exposed snails, data were also divided into two groups, with day (= light phase including time points 04:30 pm, 08:30 am and 12:30 pm) and night records (= dark phase including time points 08:30 pm, 12:30 am and 04:30 am), respectively, on which a t-test (two tailed P<0.05) was applied.

## Results

### CdMT mRNA transcription under Cd stress

**Fig 1:**
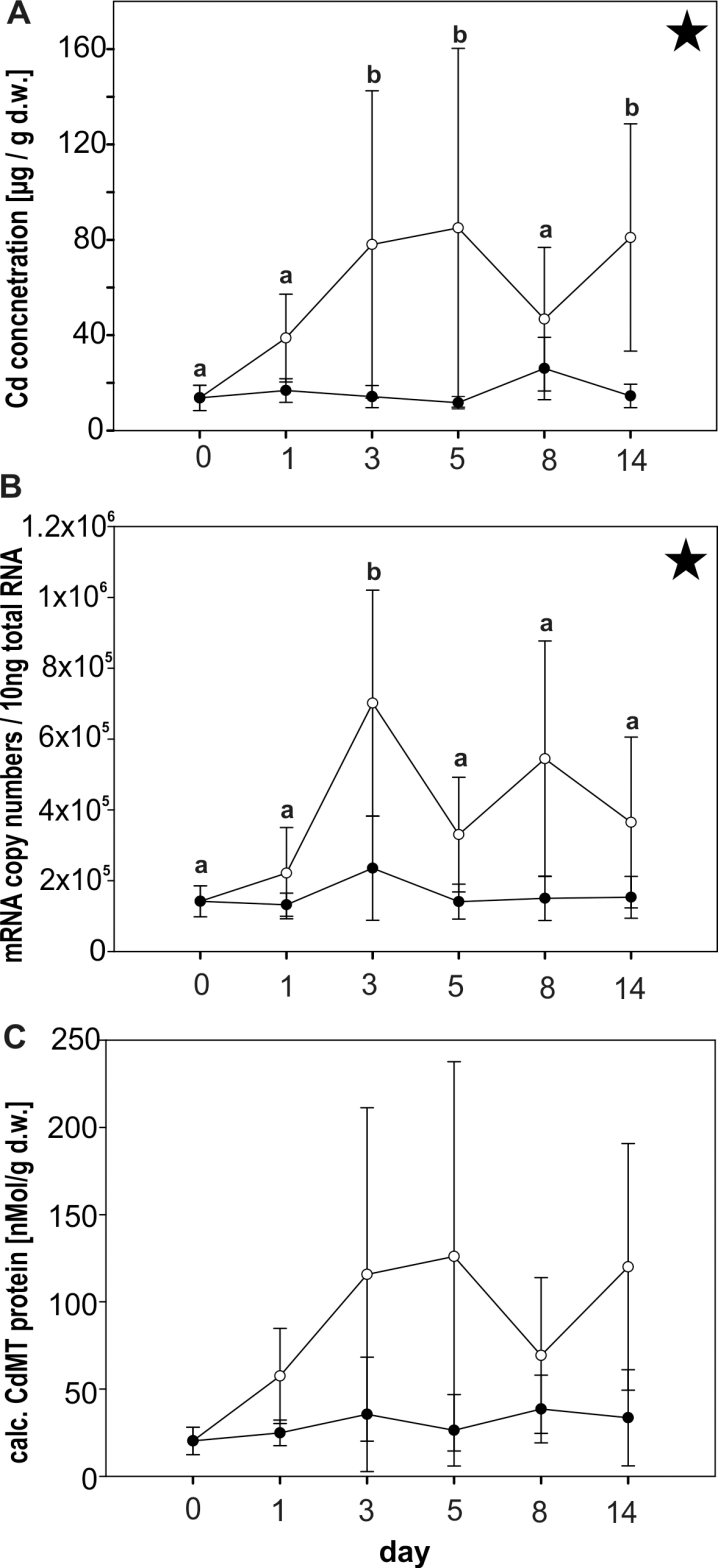
Cd accumulation and upregulation of the *CdMT* gene in *Helix pomatia* due to Cd stress. Cd concentrations (d.w.) **(A),** CdMT mRNA copy numbers **(B)** and calculated CdMT protein concentrations **(C)** of control Roman snails (black circles) and Cd-exposed individuals (white circles) were assessed over a period of 14 days. For both groups (n = 4–5) mean values and standard deviations are shown. Significant differences through experimental time and treatment by Two Way ANOVA were detected for *CdMT* mRNA copy numbers (time—P = 0.007; treatment—P = <0.001) and Cd midgut gland tissue concentrations (P = 0.042) for Cd-exposed snails (asterisks). Significant differences between single day values by multiple comparisons are indicated by different lower case letters (a,b).

Midgut gland Cd concentrations increased significantly over time (P = 0.042) in metal-exposed snails, compared to control individuals (**Fig 1A**). Cd concentrations rose rapidly until day 3, remaining at an elevated level (up to 196 μg/g d.w.), except for day 8, through the end of the experiment. In contrast, midgut gland Cd concentrations of controls remained with about 17 μg/g dry weight (d.w.) (SD ± 8) at a persistently low level.

In Cd-stressed Roman snails, a significant increase of CdMT mRNA copy numbers was observed due to metal treatment (P = <0.001, Two Way ANOVA) (**Fig 1B**). Time-dependent increase of CdMT mRNA expression (P = 0.007, Two Way ANOVA) was particularly distinct in the first days of Cd exposure, with a peak of transcription at day 3, when mRNA concentrations amounted to values between 311 000 and 1 000 000 copies/10ng total RNA. Overall, CdMT mRNA in Cd-exposed animals reached an average level of around 432 700 (SD ± 282 800) copies/10ng total RNA, compared to a consistently lower level of around 158 600 (SD ± 75 310) copies/10ng total RNA in control snails.

CdMT protein concentrations increased up to 299 nMol / g d.w. in Cd-exposed snails, compared to controls showing a permanently low level of approx. 30 nMol / g d.w. (SD ± 20) (**Fig 1C**).

### Physiological variability of CdMT expression: Influence of diurnal rhythmicity and feeding activity

#### Inherent diurnal rhythmicity

**Fig 2:**
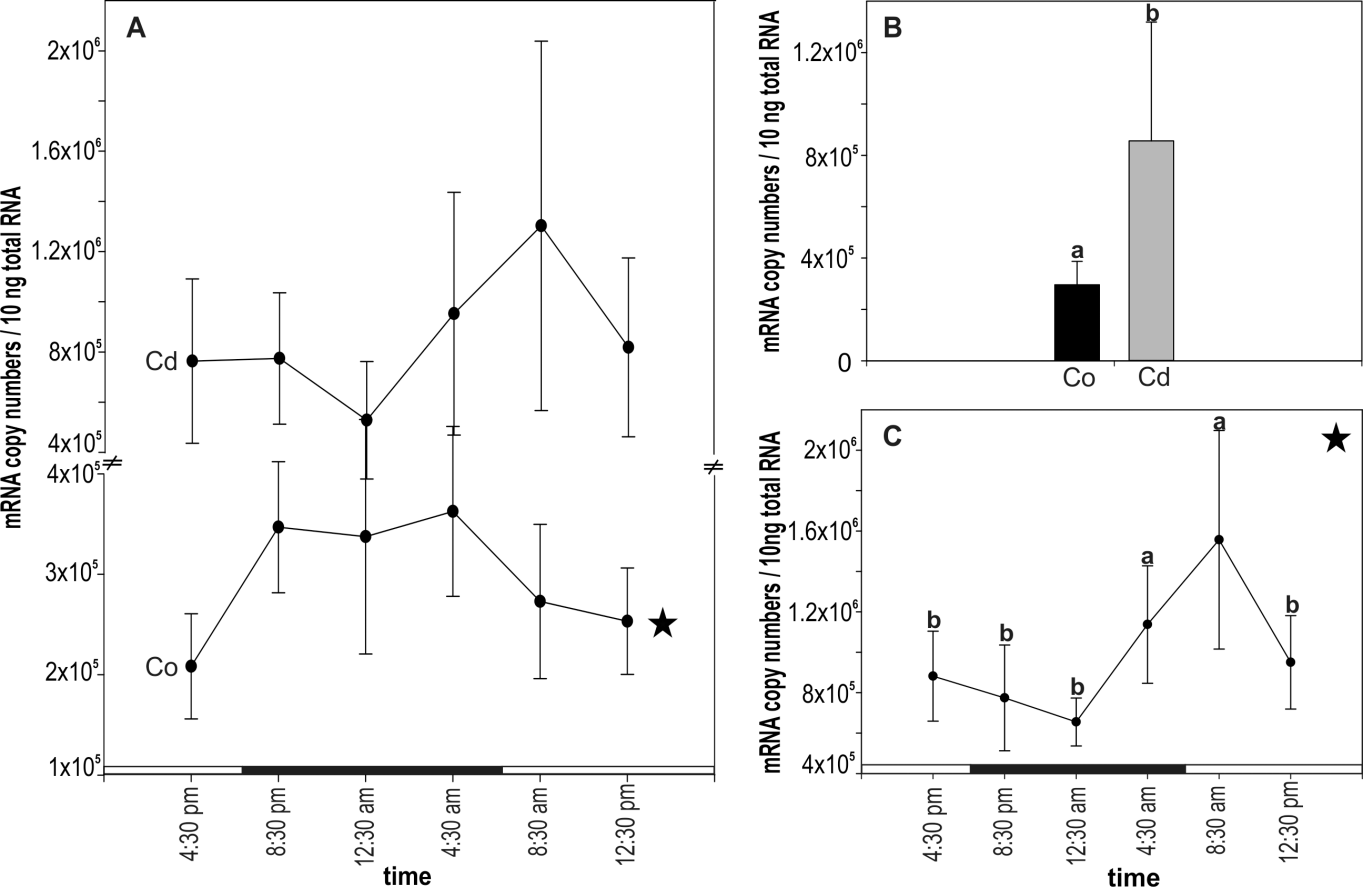
Diurnal rhythmicity of *CdMT* gene expression of control and Cd-exposed Roman snails. **(A)** CdMT mRNA copy numbers over a time span of 20 hours, including six sampling time points, in control (Co) and Cd-exposed snails (Cd) (n = 5). Significance of the curves through the diurnal cycle (Two Way ANOVA) was only observed for unexposed control snails (asterisk). No significant differences were found by multiple comparisons within the control and Cd-treated group. **(B)** Bar graph with means and standard deviation (+SD) of CdMT mRNA copy numbers between control and Cd-exposed individuals (n = 30 each). Different lowercase letters (a, b) indicate significant differences (t-test) **(C)** Time-dependent CdMT mRNA expression of Cd-exposed snails (P = <0.001) over a time span of 20 hours, shown after exclusion of non-feeders (definition see text) from the dataset (n = 3–5). The peak of CdMT mRNA copy numbers at 08:30 am was compared to all other time points. Significance of multiple comparison is indicated by different lower case letter b. Significance of Two Way ANOVA is indicated by an asterisk. The light period (long white bars) lasted from 07:00 am to 07:00 pm and dark period (long black bars) from 07:00 pm to 07:00 am.

**Fig 3:**
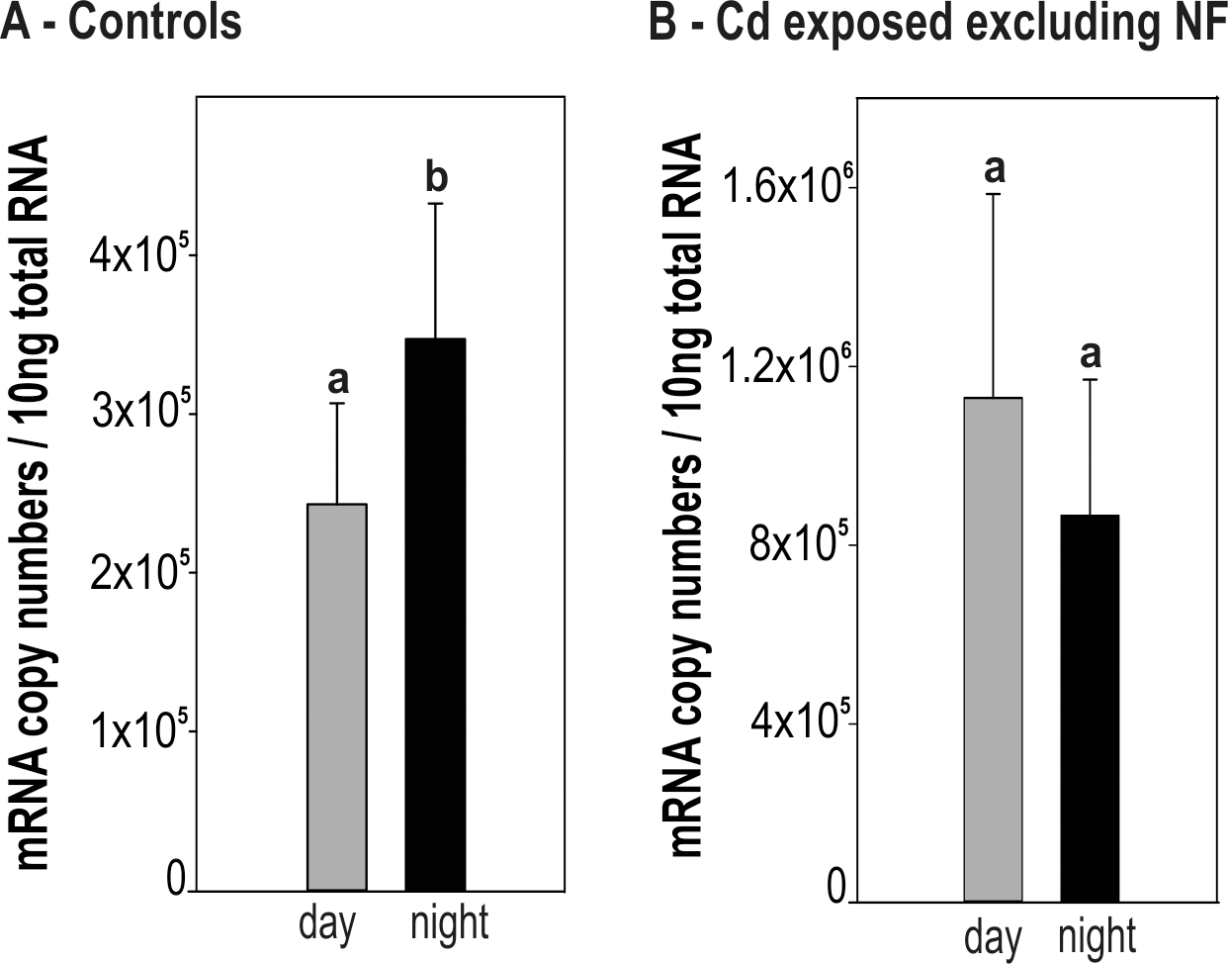
Comparison of CdMT mRNA expression between day and night in *Helix pomatia*. Bar graph with means and standard deviations (+SD) of CdMT mRNA copy numbers / 10ng total RNA of day and night-collected samples from control snails (n = 15 each) (**A**) and Cd-exposed individuals after exclusion of non-feeders (n = 12 each) (**B**) are shown. Significant differences between day (= grey bars) and night samples (= black bars) (t-test, P = 0.0008) are indicated by different lower case letters (a, b).

For unstressed control snails, the values of CdMT mRNA copy numbers appeared to depend on the time point of sampling during the diurnal cycle (P = 0.05, Two Way ANOVA). In fact, the time course of mRNA levels suggests an elevated transcription rate during the night, although a multiple single point comparison of values at each sampling time point did not show statistically significant differences (**Fig 2A**). However, when mRNA transcription values were grouped into two categories (day and night), significant differences in *CdMT* gene expression were observed between day and overnight-collected snails (P = 0.0008) (**Fig 3A**).

As expected, the level of CdMT mRNA transcription was significantly higher in Cd-exposed snails compared to control animals (P = <0.001), with an average 3-fold upregulation of CdMT transcription rate in Cd-treated snails with respect to unexposed animals (**Fig 2B**). The diurnal time course of transcription rates in Cd-fed snails, however, disappeared, and no time-dependent significance of the curve shape through the day/night cycle was found (P = 0.15) (**Fig 2A**).

#### Influence of feeding activity

The diurnal curve of Cd-exposed snails was re-drawn after exclusion of values obtained from snails which had only poorly or not fed at all on Cd-enriched salad (“non-feeders”) (**Fig 2C**), so that only “feeders” were included into the analysis. This was possible due to the fact that the feeding activity of snails during the diurnal experiment was recorded, revealing that two categories of snails could be distinguished: the category of “feeders”, representing snails, which were feeding at least two days of the five day experiment and at minimum once of these days more than half of the supplied salad leaf. All snails feeding less than described above were defined as “non-feeders”. In general, more non-feeders were found in the control group compared to the Cd-treated group, even though in control snails no significant difference was found for CdMT mRNA transcription between feeders and non-feeders (**Table 1**). It appeared that after exclusion of “non-feeders” from the Cd-exposed group, the time-dependent curve shape became significant (Two Way ANOVA, P = <0.001) (**Fig 2C**), although a comparison of single values between daytime and night snails did not show significant differences (two tailed P = 0.110) (**Fig 3B**). Instead, a peak of CdMT mRNA transcription was observed in snails collected at 08:30 am (**Fig 2C**).

**Table 1:** mRNA quantification and midgut gland Cd concentrations of controls and Cd exposed snails under consideration of differential feeding behavior.

	*mRNA copy numbers*			*Cd tissue concentrations (*μ*g/g)*		
	*# of snails*	*mean (± SD)*	*range*	*# of snails*	*mean (± SD)*	*range*
**Controls**	30	296 180.99 (± 90 965.57)	145 614.38–512 802.24	29	15.20 (± 8.09)	6.22–36.08
Controls F	18	310 982.40 (± 102 016.50)	145 614.38–512 802.24	18	16.40 (± 9.04)	7.65–36.08
Controls NF	12	273 978.87 (± 69 533.12)	161 455.12–379 541.18	11	13.24 (± 6.15)	6.22–28.45
**Cd-exposed**	30	856 605.90 (± 462 296.96)	150 892.53–2 119 320.07	25	232.80 (± 144.13)	24.12–455.98
Cd exp. F	24	997 581.65 (± 402 865.71)	513 301.29–2 119 320.07	20	274.28 (± 130.33)	24.12–455.98
Cd exp. NF	6	292 702.89 (± 126 505.53)	150 892.53–524 300.22	5	66.87 (± 30.67)	36.97–110.82

Mean values, standard deviations (± SD) and range (from the lowest to the highest values) of mRNA copy numbers and Cd concentrations in μg/g d.w. of all control and Cd-exposed individuals (n = 30 each) are reported. Individuals of both groups are separated into feeders (F) and non-feeders (NF), and the respective copy numbers and Cd tissue concentrations are shown. Due to loss of samples during tissue digestion for metal analysis, numbers of snails listed for Cd concentrations can differ from those of mRNA quantification.

#### Variability between controls and Cd-exposed snails

**Fig 4:**
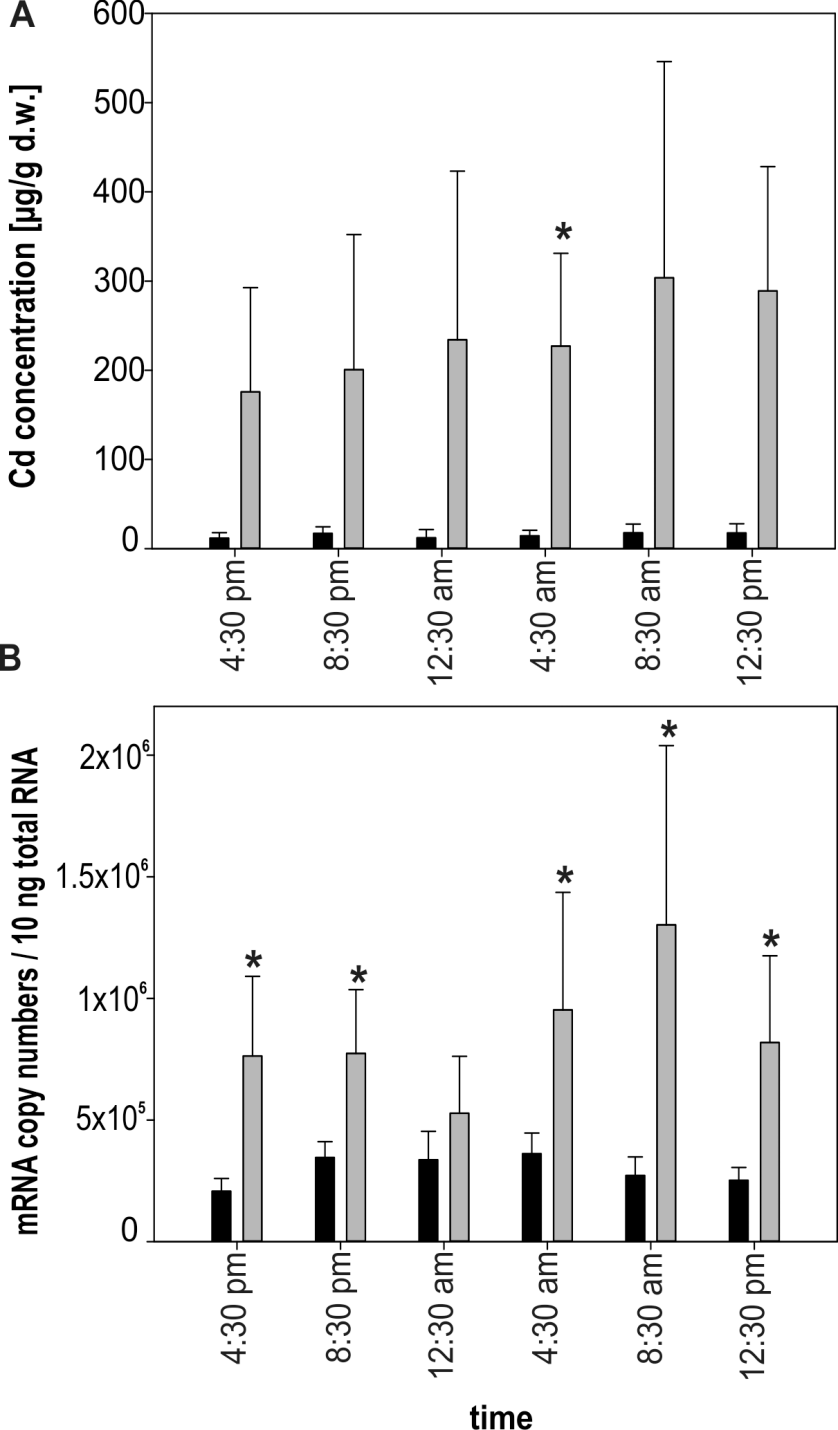
Upregulation of *CdMT* gene transcription due to Cd exposure in the Roman snail. **(A)** Bar graph of Cd concentrations (μg / g d.w.) of controls (black bars; n = 4–5) and Cd-exposed snails (grey bars; n = 3–5), including non-feeders, during the diurnal cycle. **(B)** Bar graph with means and standard deviation (+SD) of CdMT mRNA concentrations from controls (black bars; n = 5) and Cd-exposed snails excluding non-feeders (grey bars; n = 3–5) measured at six different time points of the diurnal cycle. Significant differences (P = <0.0083) between control and Cd-exposed individuals are indicated by an asterisk (multiple comparisons).

Cd concentrations through the diurnal cycle were permanently elevated in Cd-exposed snails even though significance (P = >0.0083) could be calculated only for time point 04:30 pm (**Fig 4A**) partly due to sample losses during metal measurement (**Table 1**). Overall, Cd concentrations in control snails ranged between 6.22 and 36.08 μg/g Cd d.w. (**Table 1**). In comparison, Cd concentrations of Cd-fed snails were up to 10-fold higher, with values ranging between 24.12 up to 455.98 μg/g Cd d.w. Non-feeding snails exposed to Cd showed persistently lower levels of midgut gland Cd concentrations, but still higher values compared to control individuals. The sample size at the different time points of the diurnal cycle was too low as to perform analyses by excluding non-feeders from the data set (n<3).

The situation was more complex when mRNA transcription rates were considered. For control snails, CdMT mRNA concentrations ranged between 145 000 and 512 000 copies / 10ng total RNA (**Table 1**), with no significant difference between feeders and non-feeders (P = 0.283). Therefore, statistical calculations in control group snails were performed without exclusion of non-feeders from the data set. For individuals exposed to Cd, mRNA copy numbers varied from 150 000 up to 2 100 000. Due to the dissimilar distribution of individual numbers between the two categories (feeders = 24; non-feeders = 6), no statistical test could be applied to distinguish between feeders and non-feeders among Cd-treated snails. However, low mRNA copy numbers belonged almost exclusively to non-feeding snails (**Table 1**). Therefore, analyses for Cd-exposed snails were conducted by excluding non-feeders from the data set (**see Figs 2C, 3B and 4B**). Hence, whilst Cd-exposed snails showed a significant upregulation of CdMT mRNA due to Cd exposure for three time points in the diurnal cycle (04:30 pm, 08:30 pm and 12:30 pm) when non-feeders were included into the dataset, this trend became more distinctive when non-feeders were omitted. Consequently, a significant increase of CdMT mRNA concentrations with respect to controls could now also be detected at time points 04:30 am and 08:30 am (**Fig 4B**). Only at time point 12:30 am did mRNA copy numbers of Cd-treated snails nearly approach the level of control snails (**Fig 4B**), in spite of the fact that Cd tissue concentrations in Cd-exposed snails indicated consistently elevated metal levels with respect to control individuals (**Fig 4A**).

### Cd MT gene expression during desiccation

**Fig 5:**
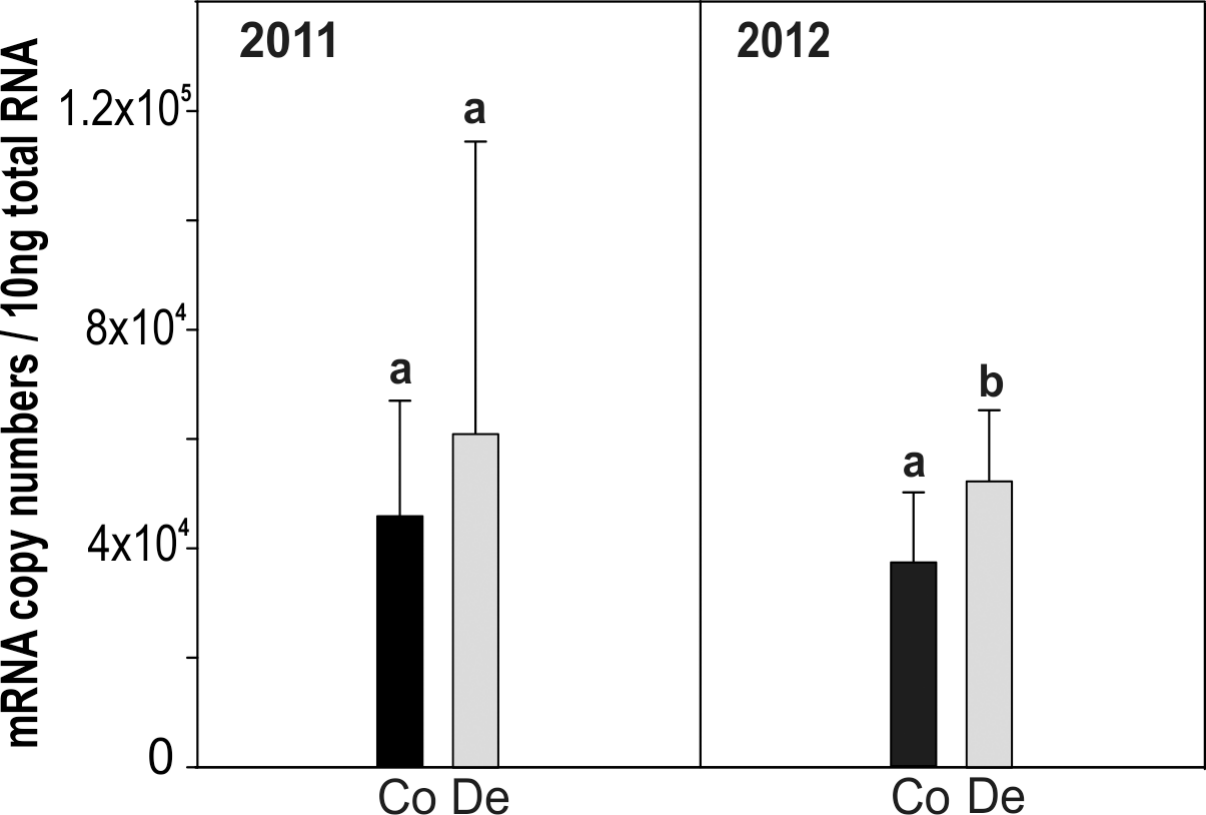
Differential *CdMT* gene expression in the Roman snail triggered by desiccation. The experiment was carried out two times, presented by the year dates 2011 (left hand side) and 2012 (right hand side). Means and +SD of CdMT mRNA copy numbers of controls (Co, black bars) and snails exposed to desiccating conditions (De, grey bars) are displayed. Significance is indicated by asterisk (P<0.05).

Exposure of snails to desiccation over a period of 21 days as of 2011 and 2012 gave rise to an increase of CdMT mRNA transcription in both experiments, although the elevation of CdMT mRNA expression values was only significant during 2012 (one tailed P<0.0496) (**Fig 5**). Midgut gland Cd concentrations for controls and desiccated snails ranged from 10 to 16 μg/g d.w.

### *CdMT* gene transcription after oxygen depletion

**Fig 6:**
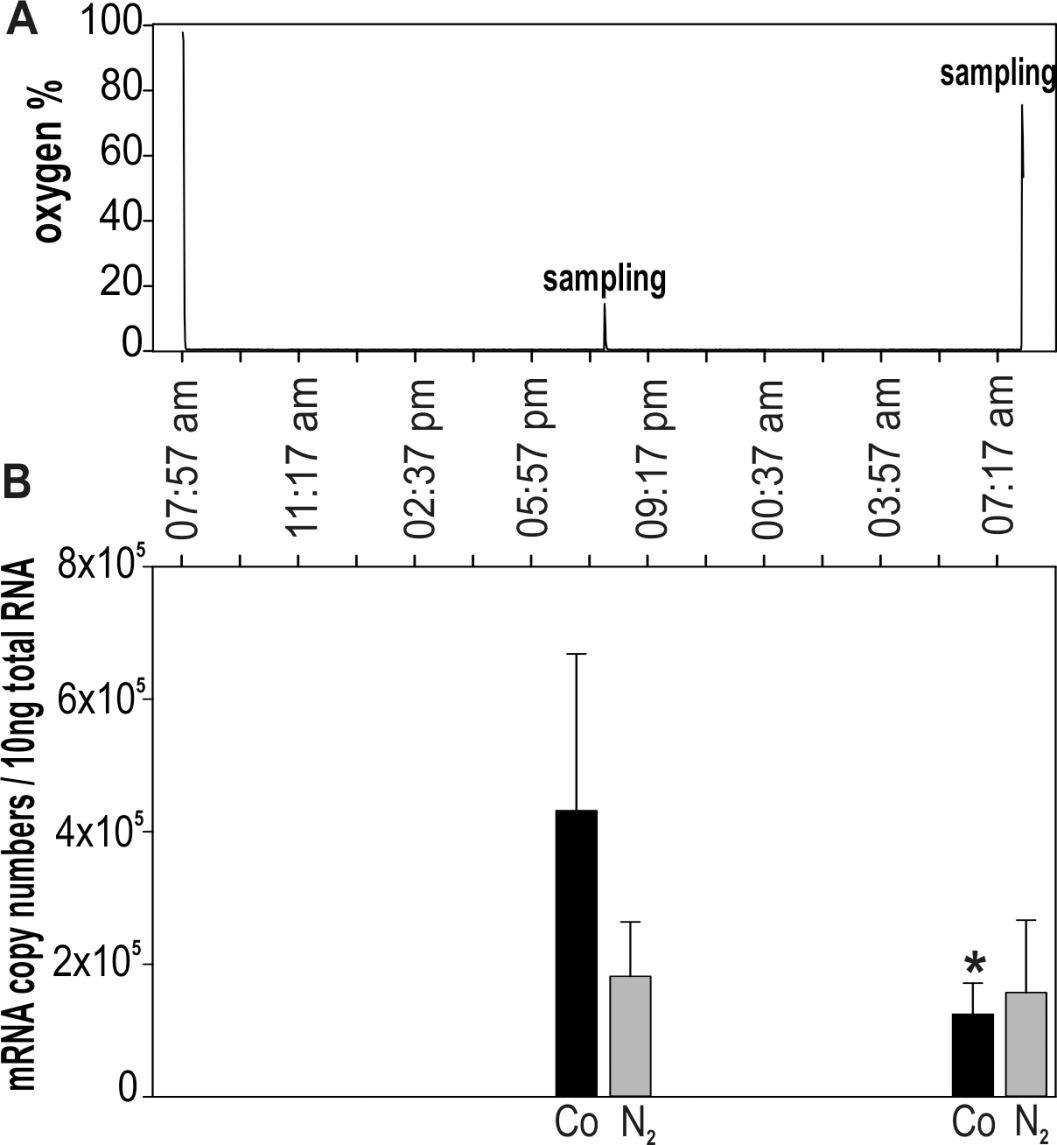
mRNA quantification of *Helix pomatia CdMT* gene transcription after exposure to oxygen depletion through 24 hours. **(A)** Plot of average oxygen concentration (%) in the atmosphere of the exposure chamber throughout the whole experiment, showing one oxygen peak due to sampling at 08:00 pm, when the oxygen level in the chamber rose up to 14% and decreased within 5 minutes under less than 1%. **(B)** Bar plot showing means and standard deviations (+SD) of CdMT mRNA copy numbers (n = 4–5) in control snails (black bars) and snails exposed to oxygen depletion (grey bars) after 12 (08:00 pm) and 24 (08:00 am) hours. The asterisk indicates a significant difference (P>0.031; Two Way ANOVA) between control snails after 12 hours and 24 hours exposure. Abbreviations: Co = control snails; N_2_ = snails exposed to oxygen depletion.

The impact of oxygen depletion on *CdMT* gene expression was tested by rearing snails under oxygen-free conditions **(Fig 6A)** for 24 hours. The mortality rate of oxygen-depleted snails over this time period increased to 45% in contrast to the much lower mortality rate of about 10% in control snails. Even though a decline of CdMT mRNA transcription was evident in oxygen-depleted snails after 12 and 24 hours, this decrease was statistically not significant (P = 0.139, Two Way ANOVA) **(Fig 6B)**.Beyond that, significantly higher CdMT mRNA copy numbers were observed in control individuals under dark conditions after 12 hours (P>0.031), compared to values in control snails sampled at daylight after 24 hours. This is consistent with the observation that CdMT mRNA transcription of control snails increases during the night (see above, **Fig 3**).

## Discussion

### Cd-dependent upregulation: differences between *CdMT* mRNA and expressed protein levels

Among all potential inducers tested so far, the non-essential metal ion Cd^2+^ has obviously the strongest upregulation capacity for the *CdMT* gene in Roman snails and some other related helicid species [17,18,19,20,31]. In fact, Cd can act as a potent inducer of MTs and MT isoforms in many other animals too [40–42]. Even though helicid *CdMT* genes and their expressed MT proteins have achieved through evolution an exceptional specificity in their response towards Cd [19,20,43], this study shows that transcriptional upregulation of the *CdMT* gene can be influenced also by other factors and stressors. In the present exposure experiment, Cd feeding gave rise to a one-phase increase of *CdMT* mRNA in exposed Roman snails within 3 days (72 hours). Concomitantly, there was a strong increase of Cd accumulation and of CdMT protein concentration in the midgut gland of metal-exposed snails in the first few days (**Fig 1**).

Due to the exclusive association of the absorbed Cd^2+^ with the expressed MT protein and the known binding stoichiometry of CdMT in the snail midgut gland [43,44], there is a strong correlation between Cd accumulation and MT concentration in this organ [18], which means that CdMT levels can directly be estimated from assessed Cd concentrations. As seen in **Fig 1A**, the time course of *CdMT* upregulation in the Roman snail midgut gland is slower than normally seen in organs of most other animal species. In vertebrates or aquatic molluscs, for example, upregulation of *MT* transcription and protein expression in liver and kidney occur within 12 to 48 hours after metal application [45–47]. The comparatively delayed transcriptional *CdMT* response in *Helix pomatia* may be due to physiological peculiarities in terrestrial snails, where the absorption of trace elements from nutrient fluids may be prolonged by the passage of food from the midgut through the branched hepatopancreatic tubular system, until reaching midgut gland cells in which *CdMT* expression is induced [28]. Moreover, animals in the present experiment were allowed to feed on the Cd-rich diet *ad libitum*, with effective metal uptake depending on individual metabolic activity and feeding cycles, which can cause a relatively large variability among single individuals (**Table 1**).

Apparently, the pattern of CdMT upregulation in our experiments differs between the gene transcriptional and the expressed protein levels. In *Helix pomatia*, CdMT mRNA transcription shows a more fluctuating pattern (**Fig 1B, Fig 4B**), compared to Cd tissue concentrations and concomitantly to CdMT protein concentrations (**Fig 1C, Fig 4A**). Additionally, CdMT protein concentrations in the metal exposure experiment follow the mRNA transcription pattern with a weaker amplitude and some time lag. We therefore suggest that after the initial transcriptional *CdMT* upregulation by Cd-dependent signaling pathways, a subsequent phase of basal CdMT transcription may take place which keeps the required protein concentration at a persistent elevated level. Once CdMT protein concentrations may have reached a maximal level capable of binding all Cd^2+^ ions present in the respective tissue, only little additional protein synthesis would subsequently be required to bind Cd^2+^ ions either released by decaying CdMT molecules [48] or entering the cell additionally through absorption from extracellular nutrient fluids.

Inconsistencies of concentrations between *MT* mRNA and protein in other animal species were explained mainly by divergent dynamics of transcriptional and translational MT regulation [47–49]. In Roman snails, the delayed pattern of the CdMT induction process may allow CdMT translation to keep in step with the transcriptional upregulation of the respective gene.

### Biorhythm and diurnal MT expression pattern

Strongest environmental cue in terrestrial snails is the light/dark cycle synchronizing behavioral rhythms with highest activity of *Helix pomatia* at dawn [5,11]. Studies of the circadian activity of *Helix pomatia* under laboratory conditions revealed an endogenous component influencing the behavior of these terrestrial snails [5], but until now it is unclear where this oscillator is situated and how it works.

It seems that genes involved in cellular stress response tend to reflect in their transcriptional activity circadian influences more likely compared to other genes [50] by oscillating expression patterns on both, protein and mRNA levels. This has been reported for heat shock proteins [34,51], hypoxia-inducible transcription factors [36,52] and many others [53–55]. It was already demonstrated, moreover, that MT genes of different organisms may be activated in a clock-related manner [56], reflecting in their activity a diurnal expression pattern [33,35,57]. This is in accordance with results derived from the present study, where an increased CdMT mRNA expression was observed in control snails at night, compared to the respective daytime levels (**Fig 3A, Fig 6B**). How diurnal MT expression may be regulated, however, still remains unclear. It could be speculated, that the diurnal expression pattern is a result of the circadian control of an important regulatory transcription factor, binding to the CdMT promoter region, or may occur through direct influence of the *CdMT* gene by core clock proteins. DNA-binding of the Heat shock factor-1 (Hsf-1), for example, follows a distinct rhythmic pattern and as a consequence, the expression of heat shock proteins is upregulated at the beginning of the dark phase, when the animal’s activity rises [34].

Environmental stressors can influence the diurnal expression pattern of proteins by interrupting their circadian rhythmicity [58,59] or establishing an oscillating expression pattern which is not present under stress-free conditions [36]. Compared to unexposed animals, snails exposed to Cd apparently show a disturbed diurnal CdMT mRNA expression pattern (**Fig 2**), with the lowest *CdMT* transcription at 12:30 am, nearly reaching *CdMT* control levels, and the highest CdMT mRNA concentration at time point 08:30 am. This seems to reflect a reversal of the diurnal *CdMT* gene expression pattern in metal-exposed snails and is also indicated by the tendentially higher *CdMT* mRNA expression values during the light period (day), even though significance in this case is not given (**Fig 3B**).

It was already demonstrated that Cd can disrupt the diurnal expression of redox enzymes and clock genes [59,60]. It can be speculated that under control conditions, regulation of the *CdMT* gene transcription may follow diurnal fluctuations of other cell components, whereas detoxification of Cd becomes essential for the animal’s survival upon metal exposure. It was hypothesized, that when glutathione levels are low due to diurnal variations, MTs may adopt the function of glutathione as a part of the antioxidant defense system of the cell [35]. In summary, all these results indicate that diurnal expression patterns of *CdMT* transcription may reflect a biological rhythmicity that could also influence heavy metal detoxification and toxicity effects. Further studies will be necessary, however, to accurately analyze the relation between circadian rhythmicity and diurnal MT expression patterns in helicid snails.

### Moderate CdMT upregulation due to desiccation

Desiccation and starvation are common physiological stressors being present in the natural habitat of terrestrial snails during summer. As a consequence, snails are able to undergo aestivation by metabolic rate depression and endure such threatening conditions [9,61]. In our experiment, snails were brought to aestivation by exposure to a prolonged period of desiccation and starvation. As seen in **Fig 5**, this has given rise to a moderate upregulation of CdMT mRNA transcription, with a significant increase of mRNA copy numbers in snails from the 2012 experiment (one-tailed P = 0.0496). The lacking significance of CdMT mRNA increase in aestivating snails of the 2011 experiment may be due to the high variability between individuals in combination with the very moderate *CdMT* gene upregulation of 1.3 to 1.4-fold only (**Fig 5**). Overall, these result are in accordance with reports of Egg et al. [30], demonstrating that CdMT protein is upregulated in *Helix pomatia* under the same desiccating laboratory conditions as applied in the present study. Furthermore, MT upregulation by desiccation has been demonstrated also in other species like the Antarctic midge [62] or in plants [63–64]. However, an impact of additional stressors, caused by metabolic rate depression, on CdMT mRNA expression cannot be excluded [30].

CdMT mRNA upregulation may in these cases be part of the general stress response, playing a role in long term survival by protection against cell damage [9,30,65]. Apart from *MT* genes, antioxidant enzyme activities and other stress-related proteins are also upregulated in aestivating snails [3,61,66]. Hence, due to depression of many metabolic processes, especially the less essential ones, during aestivation [9,65], a slightly increased CdMT upregulation may compensate for possible resulting negative effects by partially taking over tasks of these silenced biosynthetic processes in order to stabilize the cell. It is speculated, that under such circumstances (in the absence of Cd^2+^) the isoform may be loaded with Zn^2+^. As shown previously, the CdMT protein may indeed be upregulated by high concentrations of Zn^2+^ [19] possibly to avoid cell damage by excessive Zn^2+^ levels [67].

### Impact of oxygen depletion on MT expression

To overcome very low temperatures in winter, snails hibernate in self-dug earth holes, where they may also experience periods of hypoxia or anoxia due to apnoic breathing patterns [68,69]. It has already been demonstrated that MT expression can be upregulated during hypoxia or anoxia as it was shown for mammalian cells and cell systems [70], as well as for marine invertebrates, including the snail *Littorina littorea* [71], the mussel *Crassostrea gigas* [72], and the prawn *Litopenaeus vannamei* [73]. Overall, there is multiple evidence that MTs can act as oxidant scavengers, too [74].

Results of the present study indicate, however, that in *Helix pomatia*, oxygen depletion is not able to enhance CdMT mRNA expression after 12 and 24 hours of exposure to oxygen-free conditions (**Fig 6B**). Though, we are not able yet to exclude the possibility of CdMT mRNA upregulation by oxygen depletion due to some experimental limitations: Firstly, two different behavioral states of exposed individuals during the experiment were observed: Most of the snails stayed inactive and capsuled themselves, whereas some other animals remained active by crawling around. This differential behavior could have diverging effects by increasing the MT mRNA transcription variability between individuals. Secondly, upregulation of the *CdMT* gene may have happened shortly after snails were exposed to oxygen-free conditions or after the termination of the experiment. Results also may indicate a decrease of *CdMT* gene expression after 12 hours (see **Fig 6B**) which was reported for a neotropical fish species and was explained by cellular defense signaling pathways due to hypoxic stress [75].

To fully evaluate the possible impact of oxygen depletion on *CdMT* gene expression in *Helix pomatia*, however, further studies will be needed, by considering more carefully individual variabilities due to differential activity behavior patterns.

## Conclusion

This study demonstrates that Cd is the most potent inducer of *CdMT* gene expression in the Roman snail *Helix pomatia* in comparison to applied non-metallic stressors (desiccation and oxygen depletion). Furthermore, we showed that CdMT mRNA expression follows a diurnal rhythmicity and the exposure to Cd can disrupt or even may inverse this diurnal expression pattern. Further studies have to be done to clarify stress related signal transduction pathways and to reveal diurnal rhythmicity aspects of *CdMT* gene expression. Although underlaying mechanisms are not completely understood, it is clear that factors increasing MT expression variability have to be considered when using MT mRNA quantification as a biomarker for environmental stressors.

## Supporting Information

S1 Table Real Time Data. (XLSX)

S2 Table Cadmium Concentrations. (XLSX)

S3 Table Oxygen Measurement. (XLSX)

## References

[1] Wünnenberg W. Diurnal rhythms of heart rate in the snail *Helix pomatia L*. Comp Biochem Physiol A Physiol. 1991;99(3):415–7.

[2] Gomot P, Gomot L. Inhibition of temperature-induced spermatogenic proliferation by a brain factor in hibernating *Helix aspersa* (Mollusca). Experientia. 1989;45(4):349–51. 2707375 10.1007/BF01957474

[3] Hermes-Lima M, Storey JM, Storey KB. Antioxidant defenses and metabolic depression. The hypothesis of preparation for oxidative stress in landsnails. Comp Biochem Physiol B Biochem Mol Biol. 1998;120(3):437–48. 9787804 10.1016/s0305-0491(98)10053-6

[4] Ferreira-Cravo M, Welker AF, Hermes-Lima M. The connection between oxidative stress and estivation in gastropods and anurans. Prog Mol Subcell Biol. 2010;49:47–61. 10.1007/978-3-642-02421-4_3 20069404

[5] Attia J. Behavioural Rhythms of Land Snails in the Field. Biological Rhythm Research. 2004;35(1–2):35–41.

[6] Nicolai A, Filser J, Lenz R, Bertrand C, Charrier M. Adjustment of metabolite composition in the haemolymph to seasonal variations in the land snail *Helix pomatia*. J Comp Physiol B. 2011;181(4):457–66. 10.1007/s00360-010-0539-x 21136264

[7] Ansart A, Vernon P, Daguzan J. Photoperiod is the main cue that triggers supercooling ability in the land snail, *Helix aspersa* (Gastropoda: Helicidae). Cryobiology. 2001;42(4):266–73. 11748935 10.1006/cryo.2001.2332

[8] Pakay JL, Withers PC, Hobbs AA, Guppy M. In vivo downregulation of protein synthesis in the snail *Helix aspersa* during estivation. Am J Physiol Regul Integr Comp Physiol. 2002;283(1):R197–204. 12069946 10.1152/ajpregu.00636.2001

[9] Storey KB, Storey JM. Metabolic rate depression in animals: transcriptional and translational controls. Biol Rev Camb Philos Soc. 2004;79(1):207–33. 15005178 10.1017/s1464793103006195

[10] Kerney MP, Cameron RAD, Jungbluth JH. Die Landschnecken Nord- und Mitteleuropas. Hamburg, Paul Parey Verlag; 1983

[11] Blanc A (1980) Données sur les activités rythmiques comportementales de l’escargot de Bourgogne (*Helix pomatia L*.). PhD Thesis, Lyon, France, 191 pp.

[12] Mohawk JA, Green CB, Takahashi JS. Central and peripheral circadian clocks in mammals. Annu Rev Neurosci. 2012;35:445–62. 10.1146/annurev-neuro-060909-153128 22483041 PMC3710582

[13] Dallinger R, Berger B, Hunziker PE, Birchler N, Hauer CR, Kägi JH. Purification and primary structure of snail metallothionein. Similarity of the N-terminal sequence with histones H4 and H2A. Eur J Biochem. 1993;216(3):739–46. 8404892 10.1111/j.1432-1033.1993.tb18193.x

[14] Dallinger R. Metallothionein research in terrestrial invertebrates: Synopsis and perspectives. Comp Biochem Physiol C Pharmacol Toxicol Endocrinol. 1996;113(2):125–33. 8646613 10.1016/0742-8413(95)02078-0

[15] Chabicovsky M, Klepal W, Dallinger R. Mechanisms of cadmium toxicity in terrestrial pulmonates: programmed cell death and metallothionein overload. Environ. Toxicol. Chem. 2004;23(3):648–55. 15285358 10.1897/02-617

[16] Hödl E, Felder E, Chabicovsky M, Dallinger R. Cadmium stress stimulates tissue turnover in *Helix pomatia*: increasing cell proliferation from metal tolerance to exhaustion in molluscan midgut gland. Cell Tissue Res. 2010;341(1):159–71. 10.1007/s00441-010-0980-x 20480182

[17] Dallinger R, Chabicovsky M, Berger B. Isoform-specific quantification of metallothionein in the terrestrial gastropod *Helix pomatia*. I. Molecular, biochemical, and methodical background. Environ Toxicol Chem. 2004;23(4):890–901. 15095884 10.1897/03-100

[18] Dallinger R, Chabicovsky M, Lagg B, Schipflinger R, Weirich HG, Berger B. Isoform-specific quantification of metallothionein in the terrestrial gastropod *Helix pomatia*. II. A differential biomarker approach under laboratory and field conditions. Environ. Toxicol. Chem. 2004;23(4):902–10. 15095885 10.1897/03-101

[19] Palacios Ò, Pagani A, Pérez-Rafael S, Egg M, Höckner M, Brandstätter A, et al Shaping mechanisms of metal specificity in a family of metazoan metallothioneins: evolutionary differentiation of mollusc metallothioneins. BMC Biology 2011;9(4). 10.1186/1741-7007-9-4 PMC3033865 21255385

[20] Dallinger R., Berger B., Hunziker PE, Kägi JHR. Metallothionein in snail Cd and Cu metabolism. Nature (London) 1997;388(6639):237–38. 9230430 10.1038/40785

[21] Egli D, Yepiskoposyan H, Selvaraj A, Balamurugan K, Rajaram R, Simons A, et al A family knockout of all four Drosophila metallothioneins reveals a central role in copper homeostasis and detoxification. Mol. Cell. Biol. 2006;26(6):2286–96. 16508004 10.1128/MCB.26.6.2286-2296.2006 PMC1430275

[22] Fu C., Miao W. Cloning and characterization of a new multi-stress inducible metallothionein gene in *Tetrahymena pyriformis*. Protist 2006;157(2):193–203. 16621695 10.1016/j.protis.2006.02.006

[23] Baird SK, Kurz T, Brunk UT. Metallothionein protects against oxidative stress-induced lysosomal destabilization. Biochem. J. 2006;394:275–83. 16236025 10.1042/BJ20051143 PMC1386026

[24] Kanekiyo M, Itoh N, Kawasaki A, Matsuda K, Nakanishi T, Tanaka K. Metallothionein is required for zinc-induced expression of the macrophage colony stimulating factor gene. J. Cell Biochem. 2002;86(1):145–53. 12112025 10.1002/jcb.10202

[25] Blindauer C. Metallothioneins In RSC Metallobiology: Binding, Transport and Storage of Metal Ions in Biological Cells; Maret W, Wedd A. Eds.; The Royal Society of Chemistry: Cambridge, UK, 2014;2:594–653.

[26] Pérez-Rafael S, Mezger A, Lieb B, Dallinger R, Capdevila M, Palacios O, et al The metal binding abilities of *Megathura crenulata* metallothionein (McMT) in the frame of gastropoda MTs. J Inorg Biochem. 2012;108:84–90. 10.1016/j.jinorgbio.2011.11.025 22209022

[27] Berger B, Dallinger R, Gehrig P, Hunziker PE 1997. Primary structure of a copper-binding metallothionein from mantle tissue of the terrestrial gastropod *Helix pomatia L*. Biochem J. 1997;15;328 (Pt 1):219–24. 9359856 10.1042/bj3280219 PMC1218909

[28] Chabicovsky M, Niederstätter H, Thaler R, Hödl E, Parson W, Rossmanith W, et al Localization and quantification of Cd- and Cu-specific metallothionein isoform mRNA in cells and organs of the terrestrial gastropod *Helix pomatia*. Toxicol. Appl. Pharmacol. 2003;190:25–36. 12831780 10.1016/s0041-008x(03)00148-0

[29] Dallinger R, Chabicovsky M, Hödl E, Prem C, Hunziker P, Manzl C. Copper in *Helix pomatia* (Gastropoda) is regulated by one single cell type: differently responsive metal pools in rhogocytes. Am J Physiol Regul Integr Comp Physiol. 2005;289(4):R1185–95. 15905226 10.1152/ajpregu.00052.2005

[30] Egg M, Höckner M, Brandstätter A, Schuler D, Dallinger R. Structural and bioinformatic analysis of the Roman Snail Cd-Metallothionein gene uncovers molecular adaptation towards plasticity in coping with multifarious environmental stress. Mol. Ecol. 2009;18(11):2426–43. 10.1111/j.1365-294X.2009.04191.x 19457198

[31] Hispard F, Schuler D, De Vaufleury A, Scheifler R, Badot PM, Dallinger R. Metal distribution and metallothionein induction after cadmium exposure in the terrestrial snail *Helix aspersa* (Gastropoda,Pulmonata). Environ. Toxicol. Chem. 2008;27(7):1533–42. 10.1897/07-232.1 18384240

[32] Hardeland R, Coto-Montes A, Poeggeler B. Circadian Rhythms, Oxidative Stress, and Antioxidative Defense Mechanisms. Chronobiol Int. 2003;20(6):921–62. 14680136 10.1081/cbi-120025245

[33] Mocchegiani E, Giacconi R, Cipriano C, Gasparini N, Bernardini G, Malavolta M, et al The variations during the circadian cycle of liver CD1d-unrestricted NK1.1+TCR gamma/delta+ cells lead to successful ageing. Role of metallothionein/IL-6/gp130/PARP-1 interplay in very old mice. Exp Gerontol. 2004;39(5):775–88. 15130672 10.1016/j.exger.2004.01.014

[34] Reinke H, Saini C, Fleury-Olela F, Dibner C, Benjamin IJ, Schibler U. Differential display of DNA-binding proteins reveals heat-shock factor 1 as a circadian transcription factor. Genes Dev. 2008;22(3):331–45. 10.1101/gad.453808 18245447 PMC2216693

[35] Xu YQ, Zhang D, Jin T, Cai DJ, Wu Q, Lu Y, et al Diurnal variation of hepatic antioxidant gene expression in mice. PLoS One 2012 7(8):e44237 10.1371/journal.pone.0044237 22952936 PMC3430632

[36] Pelster B, Egg M. Multiplicity of Hypoxia-Inducible Transcription Factors and Their Connection to the Circadian Clock in the Zebrafish. Physiol Biochem Zool. 2015;88(2):146–57. 10.1086/679751 25730270

[37] Hinrichsen RD, Tran JR. A circadian clock regulates sensitivity to cadmium in *Paramecium tetraurelia*. Cell Biol Toxicol. 2010;26(4):379–89. 10.1007/s10565-010-9150-x 20108033

[38] Höckner M, Stefanon K, Schuler D, Fantur R, De Vaufleury A, Dallinger R. Coping with cadmium exposure in various ways: the two helicid snails *Helix pomatia* and *Cantareus aspersus* share the metal transcription factor-2, but differ in promoter organization and transcription of their Cd-Metallothionein genes. J. Exp. Zool. 2009;311A:776–87. 10.1002/jez.564 19691054

[39] Berger B, Dallinger R. Terrestrial snails as quantitative indicators of environmental metal pollution. Environ Monit Assess. 1993;25(1):65–84. 10.1007/BF00549793 24227457

[40] Geret F, Cosson RP. Induction of specific isoforms of metallothionein in mussel tissues after exposure to cadmium or mercury. Arch Environ Contam Toxicol. 2002;42(1):36–42. 11706366 10.1007/s002440010289

[41] Amiard JC, Amiard-Triquet C, Barka S, Pellerin J, Rainbow PS. Metallothioneins in aquatic invertebrates: their role in metal detoxification and their use as biomarkers. Aquat Toxicol. 2006;76(2):160–202. 16289342 10.1016/j.aquatox.2005.08.015

[42] Brulle F, Mitta G, Leroux R, Lemière S, Leprêtre A, Vandenbulcke F. The strong induction of metallothionein gene following cadmium exposure transiently affects the expression of many genes in *Eisenia fetida*: a trade-off mechanism? Comp Biochem Physiol C Toxicol Pharmacol. 2007;144(4):334–41. 17150412 10.1016/j.cbpc.2006.10.007

[43] Berger B, Hunziker PE, Hauer CR, Birchler N, Dallinger R. Mass spectrometry and amino acid sequencing of two cadmium-binding metallothionein isoforms from the terrestrial gastropod *Arianta arbustorum*. Biochem J. 1995;311(Pt 3):951–7. 7487956 10.1042/bj3110951 PMC1136094

[44] Dallinger R, Wang Y, Berger B, Mackay EA, Kägi JH. Spectroscopic characterization of metallothionein from the terrestrial snail, *Helix pomatia*. Eur J Biochem. 2001;268(15):4126–33. 11488904 10.1046/j.1432-1327.2001.02318.x

[45] Vasconcelos MH, Tam SC, Beattie JH, Hesketh JE. Evidence for differences in the post-transcriptional regulation of rat metallothionein isoforms. Biochem J. 1996;315(Pt 2):665–71. 8615845 10.1042/bj3150665 PMC1217248

[46] Tandon SK, Singh S, Prasad S, Mathur N. Hepatic and renal metallothionein induction by an oral equimolar dose of zinc, cadmium or mercury in mice. Food Chem Toxicol. 2001;39(6):571–7. 11346487 10.1016/s0278-6915(00)00167-8

[47] Paul-Pont I, Gonzalez P, Baudrimont M, Nili H, de Montaudouin X. Short-term metallothionein inductions in the edible cockle *Cerastoderma edule* after cadmium or mercury exposure: discrepancy between mRNA and protein responses. Aquat Toxicol. 2010;97(3):260–7. 10.1016/j.aquatox.2009.12.007 20045202

[48] Miles AT, Hawksworth GM, Beattie JH, Rodilla V. Induction, regulation, degradation, and biological significance of mammalian metallothioneins. Crit Rev Biochem Mol Biol. 2000;35(1):35–70. 10755665 10.1080/10409230091169168

[49] Carginale V, Scudiero R, Capasso C, Capasso A, Kille P, di Prisco G, et al Cadmium-induced differential accumulation of metallothionein isoforms in the Antarctic icefish, which exhibits no basal metallothionein protein but high endogenous mRNA levels. Biochem J.1998;332(Pt 2):475–81. 9601077 10.1042/bj3320475 PMC1219503

[50] Hurley JM, Dasgupta A, Emerson JM, Zhou X, Ringelberg CS, Knabe N, et al Analysis of clock-regulated genes in Neurospora reveals widespread posttranscriptional control of metabolic potential. Proc Natl Acad Sci U S A. 2014;111(48):16995–7002. 10.1073/pnas.1418963111 25362047 PMC4260557

[51] Tominaga H, Coury DA, Amano H, Miki W, Kakinuma M. cDNA cloning and expression analysis of two heat shock protein genes, *Hsp90* and *Hsp60*, from a sterile *Ulva pertusa* (Ulvales, Chlorophyta). Fisheries Science 2012;78(2):415–29.

[52] Egg M, Köblitz L, Hirayama J, Schwerte T, Folterbauer C, Kurz A, et al Linking oxygen to time: the bidirectional interaction between the hypoxic signaling pathway and the circadian clock. Chronobiol Int. 2013;30(4):510–29. 10.3109/07420528.2012.754447 23421720

[53] Lemberger T, Saladin R, Vázquez M, Assimacopoulos F, Staels B, Desvergne B, et al Expression of the peroxisome proliferator-activated receptor alpha gene is stimulated by stress and follows a diurnal rhythm. J Biol Chem. 1996;271(3):1764–9. 8576180 10.1074/jbc.271.3.1764

[54] Beaver LM, Klichko VI, Chow ES, Kotwica-Rolinska J, Williamson M, Orr WC, et al Circadian regulation of glutathione levels and biosynthesis in *Drosophila melanogaster*. PLoS One. 2012; 7(11). e50454 10.1371/journal.pone.0050454 23226288 PMC3511579

[55] Soni P, Kumar G, Soda N, Singla-Pareek SL, Pareek A. Salt Overly Sensitive pathway members are influenced by diurnal rhythm in rice. Plant Signal Behav. 2013; 8(7). e24738 10.4161/psb.24738 23656875 PMC3909089

[56] Bell-Pedersen D, Shinohara ML, Loros JJ, Dunlap JC. Circadian clock-controlled genes isolated from *Neurospora crassa* are late night- to early morning-specific. Proc Natl Acad Sci U S A. 1996;93(23):13096–101. 8917550 10.1073/pnas.93.23.13096 PMC24052

[57] Miura N, Yanagiba Y, Ohtani K, Mita M, Togawa M, Hasegawa T. Diurnal variation of cadmium-induced mortality in mice. J Toxicol Sci. 2012;37(1):191–6. 22293423 10.2131/jts.37.191

[58] Guillaumond F, Lacoche S, Dulong S, Grechez-Cassiau A, Filipski E, Li XM, et al Altered Stra13 and Dec2 circadian gene expression in hypoxic cells. Biochem Biophys Res Commun. 2008;369(4):1184–9. 10.1016/j.bbrc.2008.03.009 18342625

[59] Jiménez-Ortega V, Cano Barquilla P, Fernández-Mateos P, Cardinali DP, Esquifino AI. Cadmium as an endocrine disruptor: correlation with anterior pituitary redox and circadian clock mechanisms and prevention by melatonin. Free Radic Biol Med. 2012;53(12):2287–97. 10.1016/j.freeradbiomed.2012.10.533 23085516

[60] Jiménez-Ortega V, Cardinali DP, Fernández-Mateos MP, Ríos-Lugo MJ, Scacchi PA, Esquifino AI. Effect of cadmium on 24-hour pattern in expression of redox enzyme and clock genes in rat medial basal hypothalamus. Biometals. 2010;23(2):327–37. 10.1007/s10534-010-9292-6 20107868

[61] Storey KB, Storey JM. Heat shock proteins and hypometabolism: adaptive strategy for proteome preservation. Research and Reports in Biology 2011;2:57–68.

[62] Lopez-Martinez G, Benoit JB, Rinehart JP, Elnitsky MA, Lee RE Jr, Denlinger DL. Dehydration, rehydration, and overhydration alter patterns of gene expression in the Antarctic midge, *Belgica antarctica*. J Comp Physiol B. 2009;179(4):481–91. 10.1007/s00360-008-0334-0 19125254

[63] Gorantla M, Babu PR, Lachagari VB, Reddy AM, Wusirika R, Bennetzen JL, et al Identification of stress-responsive genes in an indica rice (*Oryza sativa L*.) using ESTs generated from drought-stressed seedlings. J Exp Bot. 2007;58(2):253–65. 17132712 10.1093/jxb/erl213

[64] Yang Z, Wu Y, Li Y, Ling HQ, Chu C. 2009. OsMT1a, a type 1 metallothionein, plays the pivotal role in zinc homeostasis and drought tolerance in rice. Plant Mol Biol. 2009;70(1–2):219–29. 10.1007/s11103-009-9466-1 19229638

[65] Hochachka PW, Buck LT, Doll CJ, Land SC. Unifying theory of hypoxia tolerance: molecular/metabolic defense and rescue mechanisms for surviving oxygen lack. Proc Natl Acad Sci U S A. 1996;93(18):9493–8. 8790358 10.1073/pnas.93.18.9493 PMC38456

[66] Hermes-Lima M, Storey KB. Antioxidant defenses and metabolic depression in a pulmonate land snail. Am J Physiol. 1995;268(6 Pt 2):R1386–93. 7611513 10.1152/ajpregu.1995.268.6.R1386

[67] Maret W. The Function of Zinc Metallothionein: A Link between Cellular Zinc and Redox State J Nutr. 2000;130(5S Suppl):1455–8. Review. 10.1093/jn/130.5.1455S 10801959

[68] Barnhart MC. Respiratory gas tensions and gas exchange in active and dormant land snails *Otala lactea*. Physiological Zoology. 1986;59(6):733–45.

[69] Nowakowska A, Caputa M, Rogalska J. Natural aestivation and antioxidant defence in *Helix pomatia*: effect of acclimation to various external conditions J. Mollus. Stud. 2010;76(4):354–59.

[70] Murphy BJ, Kimura T, Sato BG, Shi Y, Andrews GK. Metallothionein induction by hypoxia involves cooperative interactions between metal-responsive transcription factor-1 and hypoxia-inducible transcription factor-1. Mol. Cancer Research 2008;6:483–90. 10.1158/1541-7786.MCR-07-0341 18337454

[71] English T, Storey K. Freezing and anoxia stresses induce expression of metallothionein in the foot muscle and hepatopancreas of the marine gastropod *Littorina littorea*. J. Exp. Biol. 2003;206:2517–24. 12796465 10.1242/jeb.00465

[72] David E, Tanguy A, Pichavant K, Moraga D. 2005. Response of the Pacific oyster *Crassostrea gigas* to hypoxia exposure under experimental conditions. FEBS Journal 2005;272:5635–52. 16262701 10.1111/j.1742-4658.2005.04960.x

[73] Felix-Portillo M, Martinez-Quintana JA, Peregrino-Uriarte AB, Yepiz-Plascencia G. The metallothionein gene from the white shrimp *Litopenaeus vannamei*: characterization and expression in response to hypoxia. Mar Environ Res. 2014;101:91–100. 10.1016/j.marenvres.2014.09.005 25299575

[74] Ruttkay-Nedecky B, Nejdl L, Gumulec J, Zitka O, Masarik M, Eckschlager T, et al The role of metallothionein in oxidative stress. Int J Mol Sci. 2013;14(3):6044–66. 10.3390/ijms14036044 23502468 PMC3634463

[75] Hauser-Davis RA, Bastos FF, Dantas RF, Tobar SA, da Cunha Bastos Neto J, da Cunha Bastos VL. et al Behaviour of the oxidant scavenger metallothionein in hypoxia-induced neotropical fish. Ecotoxicol Environ Saf. 2014;103:24–8. 10.1016/j.ecoenv.2014.01.015 24561243

